# Frontal Sinus Patency after Extended Frontal Sinusotomy Type III

**Published:** 2016-09

**Authors:** Mansour Hajbeygi, Ali Nadjafi, Amin Amali, Babak Saedi, Seyed Mousa Sadrehosseini

**Affiliations:** 1 *Department of Otorhinolaryngology, Khatamolanbia Hospital, Zahedan University of Medical Sciences, Zahedan, Iran.*; 2 *Department of Otorhinolaryngology, Medical school, Tehran University of Medical Sciences, Tehran, Iran.*; 3 *Department of Otorhinolaryngology Head Neck Surgery, Valiasr Hospital, Imam Khomeini Hospital Complex, Tehran, Iran.*

**Keywords:** Draf, Frontal sinus, FESS, Polyposis, Sinusitis, Surgery

## Abstract

**Introduction:**

The surgical management of chronic frontal sinus disorders remains a challenge for rhinologists. The aim of this study was to evaluate the result of Draf III in a series of patients who underwent this procedure.

**Materials and Methods:**

Twenty patients were included in this study. Demographic data, history of prior surgery, asthma, aspirin sensitivity and Lund–Mackay score were recorded. A visual analog scale was used for frontal-related symptoms. Patients were followed for a mean duration of 17.5 months and the patency of the frontal sinus ostium was closely monitored.

**Results:**

Fifteen patients with chronic frontal sinusitis, two patients with mucoceles, two with malignancy, and one with osteoma underwent Draf III. The mean symptoms score significantly decreased from 5.9 to 3. No ostial closure was seen in the follow-up period. Among 15 patients with chronic frontal sinusitis, 12 had patent ostia of whom three had significant stenosis. All patients with mucocele and osteoma had patent ostia in the follow-up period but patients with sinonasal malignancy showed significant stenosis.

**Conclusion:**

Draf III frontal sinusotomy is successful in alleviating patient symptoms and the frontal sinus neo-ostium will remain patent in long-term follow-up of most patients. Revision surgery will be required in some cases, which seems to be related to the nature of the underlying chronic sinus diseases.

## Introduction

Despite considerable advances in surgical techniques and instruments, the surgical management of chronic frontal sinus disorders remains a serious challenge for rhinologists due to the complex anatomy of the frontal sinus, difficulty in accessing it through the nose, and restenosis after the surgery.

Before the introduction of endoscopy in sinus surgery, complex cases of the frontal sinus were managed through the anterior wall using an osteoplastic flap approach (1,2). This technique was relatively successful in controlling chronic and difficult-to-treat frontal sinus pathologies, but was invasive with adverse effects and failure in 6–25 percent of patients (3,4).

Different endoscopic techniques have been introduced for performing frontal sinusotomy. Draf categorized them into four techniques (5). In the Draf III (endoscopic modified Lothrop) procedure, the floor of both frontal sinuses along with the frontal intersinus septum and upper septum are removed and a new wide median drainage is created. In this way, maximal exposure of the frontal sinus including its lateral parts can be provided.

Although the optimal technique for patients with frontal sinus pathologies remains a controversial topic, different conditions are described as indications for Draf III, including frontal sinus mucocele, chronic frontal sinusitis, massive nasal polyposis, neo-osteogenesis or adhesion following surgery, massive fungal infection, inverted papilloma, and osteoma.

Stenosis or closure of the frontal sinus ostium and surgical failure is a main concern for rhinologists who perform endoscopic modified Lothrop procedure (EMLP). The aim of this study was to follow and evaluate the result of Draf III in a series of patients who underwent this procedure.

## Materials and methods

The study protocol was approved by the institutional review board of Tehran University of Medical Sciences. 

Detailed information about the study was provided to the participants in writing, and informed consent was obtained from each participant. All aspects of the study were conducted according to the Declaration of Helsinki. Patients who were referred to Imam Khomeini Medical Center (a tertiary academic referral hospital affiliated with Tehran University of Medical Sciences) between April 2010 and December 2012 were enrolled in the study. In addition to demographic data, we evaluated the duration of sinusitis, history of asthma, and aspirin sensitivity in every patient.

Symptoms including forehead pain and congestion as well as surgical indications were recorded. All patients with a diagnosis of nasal polyposis received maximal medical treatments (i.e., one puff of fluticasone nasal spray twice daily plus amoxicillin clavulanic acid (625 mg tablet) three times daily for at least 1 month) before surgery. All patients also underwent complete nasal examination, including nasal endoscopy to determine the presence of polyps, septal deviation, and other anatomical variations. All patients underwent axial and coronal computed tomography (CT) scanning before surgery, and the images were scored according to the Lund–Mackay system prior to the procedure. All images were assessed and reported by the same radiologist. Patients were candidates for enrollment if they had advanced frontal sinus disease. Patients lost to follow-up were excluded from the analysis.

All procedures were conducted using general anesthesia and neuronavigation (Parsiss Co. Tehran, Iran). Before surgery, oral antibiotics and oral prednisolone (0.5 mg/kg) were administered to patients with chronic frontal sinusitis for at least 1 week. All patients were hospitalized for at least 24 hours after surgery and were then discharged if there were no concerns. Postoperatively, all patients received normal saline and oxymetazoline nasal sprays for 1 week. The only prescribed analgesic was acetaminophen tablets. Patients with chronic sinusitis with polyps continued treatment with inhaled nasal corticosteroid spray (budesonide) twice daily, subject to change depending on endoscopic findings, and nasal saline douche twice daily for at least 6 months. Frequent endoscopic debridement was carried out at least 3 months after surgery to induce and maintain a normal frontal sinus neo-ostium with a uniform method in all patients. The first debridement was performed after 1 week and continued after 1 and 3 months. Forehead pain and congestion were used to calculate a subjective frontal sinus-related symptom score at two timepoints: before surgery, and at the time of the last follow-up. In addition, endoscopic evaluation of the frontal sinus neo-ostium was performed. Perioperative complications including cerebrospinal fluid leak (CSF) leak and bleeding were recorded. All evaluations were conducted by one of the authors using the same method. Data were analyzed using the Statistical Package for the Social Sciences (SPSS) version 11.5 for Windows (SPSS Inc, Chicago, Illinois, USA). An analysis of variance (ANOVA) and t-test were used to evaluate pre- and post-operative quantitative data. Moreover, the Chi-square test was used to evaluate descriptive data. Furthermore, repeated measure ANOVA with post hoc analysis was used to evaluate quantitative variations among groups. Values were assessed using descriptive statistical methods (mean ± standard deviation [SD]). The results were considered significant if p-values were less than 0.05. 

## Results

Twenty-five patients were enrolled in this study, five of whom were lost to follow-up. Twenty patients in total are therefore included in this analysis. The mean follow-up period was 17.5 months. Seventeen (85%) patients were male and three (15%) were female. The mean age of the patients was 40.4 years (range, 17–70 years) and half of the patients were aged 30–50 years. There was history of prior surgery in 75% of the patients. Samter’s triad was reported in two patients.

Draf III indications were chronic frontal sinusitis in 15 patients (75%), mucocele in two (10%), malignancy in two (10%), and osteoma in one (5%) patient. All patients with chronic frontal sinusitis had pansinusitis with polyps. Three patients had a history of asthma with and without aspirin sensitivity, and there was evidence of eosinophilic mucin sinusitis in six patients based on CT appearance and observing allergic mucin during surgery. Both patients with frontal sinus mucoceles had a history of head trauma and presented with unilateral and bilateral proptosis (Table. 1).

**Table 1:** Surgical indications, pre- and post-operative symptoms score, Lund–Mackay score and mean follow-up of patients

**Pathology**	**Number**	**Preoperative symptoms score (SD)**	**Post-operative symptoms score (SD)**	**P-Value**	**Lund-** **Mackay** **Score**	**Mean** **follow-up** **(Months)**
Chronic Frontal Sinusitis	15	5.8(3.6)	0.9(1.4)	<0.001	19	18.9
- Asthma	3	3.3(5.8)	1(1.7)	0.423	17.3	30
-Eosinophilic	6	5.5(3.3)	1(1.7)	0.04	20	19
Mucocele	2	5(4.1)	0	0.126	8	15
Osteoma	1	7	0		2	8
Sinonasal Malignancy	2	7.4(0.7)	3(1.4)	0.07	12	14

Two patients with sinonasal malignancy entered study. The first patient with a prior history of radiation therapy and surgery for undifferentiated carcinoma underwent endoscopic craniofacial resection and needed Draf III as part of the procedure. The second patient with prior surgical excision of sinonasal hemangiopericytoma presented with frontal sinus opacification on the follow-up CT scan. The Draf III procedure was performed to explore frontal sinus and establish a drainage pathway.

The mean symptom score (0–10) before the surgery was 5.9 (2.7–9.1), decreasing significantly to 3 (1.5–4.5) after the procedure (P= 0.001). Symptom scores among patients with chronic frontal sinusitis were 5.8 (SD=3.6) and 0.9 (SD=1.4) before and after the surgery, respectively (P<0.001). Overall,12 patients(60%)were asymptomatic. Partial improvement was noted in six patients (30%), while two patients (10%) reported either no improvement in symptoms or developed new symptoms. One patient still had forehead congestion without pain after surgery. Imaging showed recurrence of eosinophilic mucus chronic rhinosinusitis (CRS) and revision surgery was suggested. The other patient developed moderate forehead pain after surgery. Post-operative endoscopy showed an open frontal sinus ostium which was obstructed by a polyp.

Table 2 shows the endoscopic status of the frontal sinus ostium after surgery. The frontal sinus ostium was considered open when its size was at least 5 mm. In these cases, the frontal sinus could be endoscopically examined through the patent ostium. Its mucosa may be normal or hypertrophied. In some cases, polyps obstructed the ostium, but after debridement it was found that the frontal sinus ostium was open and a suction cannula could easily touch the sinus boundaries. When the frontal sinus ostium was smaller or could not be visualized, we differentiated between significant stenosis and obstruction. When there was total opacification of the frontal sinus with no air on post-operative paranasal sinus CT scan, the ostium was considered totally blocked. 

**Table 2:** Status of frontal sinus ostium after Draf III frontal sinus surgery based on diagnostic nasal endoscopy and paranasal sinus CT

**Pathology**	**Patent with** **normal mucosa**	**Patent with** **hypertrophied** **mucosa**	**Patent but** **obstructed with** **polyp**	**Significant** **stenosis**	**Ostial** **closure**
Chronic Frontal Sinusitis	4	3	5	3	0
- Asthma	0	2	1	0	0
-Eosinophilic	2	1	2	1	0
Mucocele	2	0	0	0	0
Osteoma	1	0	0	0	0
Sinonasal Malignancy	0	0	0	2	0

**Fig 1:**
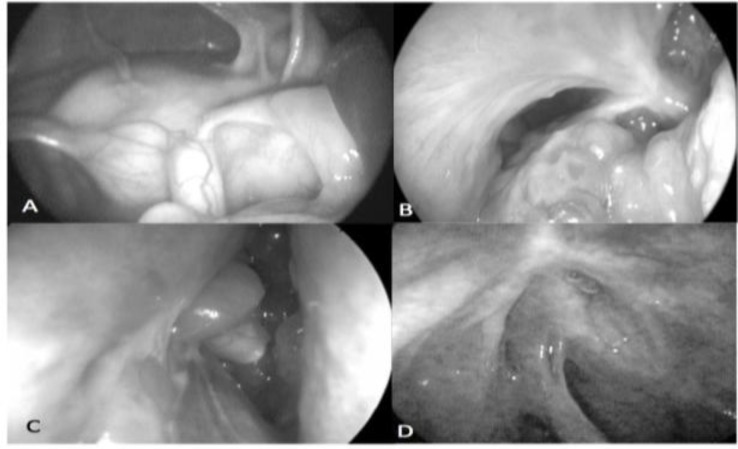
Endoscopic description of frontal sinus ostium after surgery: A) Patent with normal mucosa; B) Patent with hypertrophied mucosa; C) Patent obstructed with polyp; D) Significant stenosis

Otherwise, the presence of air in the frontal sinus indicated severe stenosis with persistence of some mucociliary transport which could prevent mucocele formation and require revision surgery in the future (Fig. 1).

Among 15 patients with chronic sinusitis with polyps, four had patent frontal sinus ostia with normal mucosa. These patients had two previous surgeries on average, but no history of asthma was noted. In three of the 15 patients, the mucosa of the frontal sinus was hypertrophied. One patient, with Samter’s triad, had undergone six previous operations, including two failed osteoplastic techniques. After 18 months, she had mild pain and congestion in the forehead. The second patient had had previous surgery and presented with exophthalmos and was diagnosed with eosinophilic mucus CRS. The patient was asymptomatic during follow-up. The final patient also had Samter’s triad and was asymptomatic during the year of follow-up. In five of the 15 patients, the frontal sinus ostium was open and a suction cannula could freely enter the sinus space, but it could not be visualized and was regarded as “obstructed” due to the presence of polyps and severe edema. Most (60%) of these patients did not have frontal sinus-related symptoms. Significant stenosis was noted in three of 15 patients. None of these patients had frontal sinus-related symptoms and all had a history of previous surgery. Frontal sinus ostial closure with accompanying total opacification and mucocele formation did not develop in any patients with CRS and polyps.

In two patients with frontal mucocele and a history of head trauma, the sinus ostia were open postoperatively with normal mucosa and the patients were asymptomatic. One patient underwent frontal sinus osteoma removal through the Draf III approach. After 8 months of follow-up, the frontal sinus ostium was patent and the mucosa was normal. Both patients with a history of sinonasal malignancy and radiotherapy had severe stenosis of the frontal sinus ostia. One patient had mild symptoms and the other complained of severe forehead pain.

## Discussion

Frontal sinus disorders and their management are still a challenging issue in rhinology. Removing whole pathology from the frontal sinus and maintaining its function in the long term are objectives of any surgical approach. Frontal sinus expansion and its anterior position hinders adequate access to the sinus to remove the lesions. Meanwhile, loss or severe damage to the frontal sinus ostial mucosa may lead to stenosis or complete closure and compromise mucociliary transport in the sinus (6).

Before the endoscopic era, the osteoplastic flap approach with obliteration of the frontal sinus was the gold standard care for chronic frontal sinus disorders. This approach, when compared with endoscopic techniques, required longer hospitalization and was accompanied by swelling and ecchymosis of the head and face and severe pain after surgery (2,7). Its failure rate was approximately 10–15% and complications were reported in 65.8% of cases. The rate of mucocele formation in the first 2 years after surgery was 9.4% (8,9). Moreover, the signs of failure could take more than 10 years after surgery to become evident (8).

Draf III/EMLP is the most extended endoscopic approach to the frontal sinus which has almost replaced the osteoplastic approach (9). It does not require external incision and has shorter hospitalization and lower costs. It creates a wide frontal sinus neo-ostium which enables close examination of the sinus and relieves the need for repeated post-operative magnetic resonance imaging (MRI) scans. The frontal beak is removed by a drill, so it is possible that the neo-ostium becomes stenotic or closed. These patients require regular and long-term follow-up after surgery and may require revision intervention.

Twenty patients in a 27-month period were followed for a mean duration of 17.5 months. Most of the patients were male, with a mean age of 40.4 years. Eighty percent of the patients had a history of previous surgery (mean, 2.5 times) and 75% of the patients had CRS with polyps. Ting JY *et al.* reported 204 patients over 16 years with a mean follow-up duration of 10.2 years. Surgical indication was CRS with/without polyps in 76.4% of cases (10). A study conducted by Shirazi MA* et al.* included 97 patients who were followed for 1.5 years. In this study, 94% of the patients had chronic frontal sinusitis and 90% had polyps (11). Georgalas et al. studied 122 patients with 33 months of follow-up in a 9-year period (12). Tran KN et al. reported on 229 patients with a mean follow-up of 45 months (7).

As most of our patients had chronic sinusitis with polyps, we focused on frontal-related symptoms (forehead pain and fullness). The mean symptom score was 5.9 (SD= 3.2) and 3 (SD=1.5) before and after surgery, respectively (P=0.001). Sixty percent of the patients were completely asymptomatic, 30% noted partial improvement and 10% reported no response or developed new symptoms. In the study by Tran KN et al., 47% of the patients were completely asymptomatic, and 27% had mild symptoms, 18% had moderate and 8% had severe symptoms after surgery. Facial pain, nasal obstruction, anosmia, and anterior and posterior nasal discharge were given as key symptoms, scaling from 1 to 5. All patients noted an improvement and none reported worsening of symptoms (7).

We proposed a new way to describe the endoscopic appearance of the frontal neo-ostium after the surgery. We used the term ‘patent’ when the size of the ostium was at least 5 mm and it was possible to have access to the frontal sinus *per se*. In some cases, polyps obstructed the ostium. Only after debridement, it was clear that the ostium was open and a suction cannula could move inside the sinus. Significant stenosis was considered when the ostium was smaller and sometimes looked like closure. The presence of air in the frontal sinus on PNS CT was taken for the presence of mucociliary transport and less chance of mucocele formation. Closure of the frontal sinus ostium meant total obstruction which required revision surgery in the future.

In our study, the frontal neo-ostium was patent in 15 cases (75%) and significantly stenosed in five cases (25%). In five cases, the frontal neo-ostium was open but obstructed with polyps. Ting JY* et al.* reported obstructed neo-ostia in 16.7% and stenosed neo-ostia in 29.9% of cases (10). Shirazi MA reported a 100% patency rate (11). The incidence of patent ostium (fully or partly) in a study by Georgalas *et al.* was 85% (12). In the study by Tran KN* et al.*, the frontal patency rate was 97% (221/229). It was occluded by polyps in six patients and was stenotic in two cases (7). No patients in our study underwent revision surgery. Two patients with recurrent fungal infection were recommended to undergo surgery for persistent symptoms. In the study by Ting JY* et al.*, nearly 30% of the patients required revision frontal sinus surgery over almost 10 years of follow-up and 10.8% required frontal obliteration. Sixty-one percent of the failures occurred in the first 2 years after surgery (10). Shirazi MA et al. reported an incidence rate of 23% of revision surgery for refractory frontal sinusitis (11). Georgalas *et al.* reported a revision surgery rate of 32%, of which 82% occurred in the first 2 years after surgery. Frontal sinus obliteration was performed in 7.4% of cases (12). Nearly 5% (12/229) of the patients in the study by Tran KN *et al.* required revision EMLP with good outcomes. The frontal sinus neo-ostium was open in four cases, obstructed with polyps in six cases, and stenosed by osteogenesis in two cases (7).

It seems the rate of revision surgery depends on multiple factors. As most of the patients in these studies suffer from chronic frontal sinusitis, debridement of the frontal sinus neo-ostium area, which in most cases remains patent, is essential. Some clinicians prefer to intervene earlier in the operation room and some are reluctant to hospitalize patients.

Due to our small sample size, we were not able to analyze factors affecting the results of surgery. Ting JY et al*.* noted that the incidence of revision surgery in patients with tumor or mucocele was much higher (five and three times respectively) than in patients who underwent surgery for a diagnosis of CRS (10). Shirazi MA et al. underlined the importance of aspirin sensitivity, nasal polyposis, asthma and allergy in surgical failure (11). Georgalas et al. found a weak association between allergy and frontal outflow tract obstruction (12). Allergic fungal sinusitis was noted as a significant risk of failure in the study by Tran KN et al. (7).

In the current study, no ocular complication or CSF leak was observed. Ting JY et al. reported four cases of CSF leak (1.9%) that were repaired primarily with good outcomes (10). Shirazi MAet al. reported one case of CSF leak (1%). Georgalas et al. and Tran KN et al. reported no complications. Our major limitation of this study was the inadequate number of cases. Additionally, the short duration of follow-up could affect the final results. Otherwise, this preliminary report is promising and the results are in accordance with other similar investigations in the medical literature.

## Conclusion

Draf III/EMLP frontal sinusotomy is the most advanced endoscopic approach to frontal sinus that could be used to address difficult-to-treat frontal sinus cases. It has replaced frontal sinus obliteration in majority of cases. It seems that this technique is quite successful in controlling patient symptoms and that the frontal sinus neo-ostium will remain patent in the long term in the majority of patients. Some patients require revision surgery which mostly relate to the unresolved nature of these chronic sinus diseases.
